# Urban nature-based solutions as public-health infrastructure: a scoping review of implemented interventions

**DOI:** 10.1007/s00114-026-02139-7

**Published:** 2026-08-03

**Authors:** Damianos Kalpakidis, Georgios Efthimiou, Constantina Skanavis, Ioannis Τ. Papadas

**Affiliations:** 1 Department of Public & Community Health, School of Public Health, University of West Attica, Athens, 11521 Greece; 2 Department of Agribusiness and Supply Chain Management, School of Applied Economics and Social Sciences, Agricultural University of Athens, Thiva, 32200 Greece; 3 Research Unit of Environmental Education & Communication, Department of Public & Community Health, School of Public Health, University of West Attica, Athens, 11521 Greece

**Keywords:** Urban nature-based solutions, Green infrastructure, Blue infrastructure, Public health, Environmental exposure mitigation, Health equity, Scoping review

## Abstract

Urban nature-based solutions (NBS) are increasingly promoted as tools to make cities healthier, more climate-resilient and more equitable, but most reviews still rely on observational studies of greener versus less green neighbourhoods. This scoping review examined implemented urban NBS and blue-green infrastructure (BGI) (such as parks, greenways, blue-green corridors, vegetated barriers and wetlands) that were evaluated using quantitative designs measuring either health outcomes or health-relevant environmental exposures. Findings were organised using the three-pathway model of Markevych et al. (2017): restoration, instoration or behavioural activation, and mitigation of environmental exposures. Primary studies with evaluative or quasi-experimental design were searched in PubMed, including randomized trials, controlled before-after, difference-in-differences, and controlled field measurement. Eleven studies were included, results were summarized narratively, and risk of bias was assessed qualitatively using design-specific domains. Evidence from recent field and quasi-experimental studies reveals three complementary pathways through which NBS influence health. First, structured exposure programs in urban forests and parks produced consistent improvements in stress, mood, anxiety and, in some cases, sleep. Second, landscape-scale interventions such as greenways, canal restorations and large new parks were associated with increases in walking and cycling, better perceived safety and, at city level, narrower mortality or childhood overweight gaps between areas. Third, NBS designed for environmental protection reduced heat, airborne pollutants and potentially pathogenic microbial loads, demonstrating a protective function. Τhese findings indicate that urban NBS can operate as active public-health infrastructure. However, the evidence base remains small, with limited long-term follow-up and few studies linking environmental change directly to clinical outcomes. Future work should integrate health monitoring into NBS design to support evidence-based urban policy. Limitations include restriction of the search to PubMed and the absence of direct clinical outcome measurement in four included studies.

## Introduction

In recent years, urban health has started to look less like a medical issue and more like a design problem. Urban residents are exposed to elevated levels of air pollution, traffic noise, and thermal stress, while physical inactivity and psychosocial strain contribute to the global burden of non-communicable diseases (Rajagopalan et al. 2024; Arriazu-Ramos et al. 2025). Cities now face the challenge of creating environments that promote health while adapting to climate change, managing pollution, and addressing social inequality (Rajagopalan et al. 2024; Arriazu-Ramos et al. 2025). This has ensured that urban planning has been given a focus in the public-health discourse.

Not all people in the city perceive the city in a similar manner. The inequalities of the society and spaces define the beneficiaries of urban green and those who experience vulnerabilities to poor environments. Socioeconomically disadvantaged populations often have poorer availability and accessibility to urban green and blue spaces, which may restrict opportunities for restorative contact with nature and contribute to existing health inequalities (Schüle et al. 2019; Rodriguez Castañeda et al. 2024). These disparities are further exacerbated by climate change, particularly among vulnerable populations and communities with limited adaptive capacity (Parsons et al. 2025). Understanding and addressing these inequalities is essential for designing healthier and more climate-resilient cities (Arriazu-Ramos et al. 2025). In this scenario, nature-based solutions (NBS) have become a new promising urban approach to address environmental and health issues at the same time.

Characterized by the European Commission as the interventions that cooperate with nature to provide societal values, NBS are intended to provide biodiversity, climate resilience, and human well-being in a holistic manner, as defined by the International Union of Conservation of Nature (IUCN). In this review, we use the term NBS/BGI to refer to both intentionally designed nature-based solutions and the broader category of blue-green infrastructure (BGI) — which includes parks, street trees, green roofs and wetlands — when the original included study does not explicitly apply the NBS label. Part of urban NBS, including parks, street trees, forests, blue-green corridors, green roofs, and wetlands, can perform ecological tasks and at the same time provide public space, cooling, and aesthetics. Integrating ecological and social aims, they are the transition to the multi-functional urban system of isolated projects on greening (Price 2021; Sowińska-Świerkosz and Garcia 2022; Viti et al. 2022; Athokpam et al. 2024). Green-blue infrastructure (BGI) is the broader infrastructural category that includes parks, street trees, greenways, green roofs, blue spaces, wetlands and vegetated barriers, whether or not the intentionality and co-benefit requirements of NBS are explicitly stated. Because the empirical literature does not always label implemented green or blue projects as NBS, we refer to NBS/BGI when the intervention has the characteristics of an urban nature-based or green-blue intervention but the original study uses different terminology.

The conceptual basis for linking urban nature and health is well established. Markevych et al. (2017) proposed three main pathways by which greenspace may influence health: mitigation of environmental harms, restoration of depleted psychological and physiological capacities, and instoration or building capacities through physical activity, social contact and other salutogenic resources. Bratman et al. (2019) similarly emphasized that nature experience can contribute to cognitive functioning, emotional wellbeing and broader mental-health outcomes, while also noting the need for stronger causal evidence. These frameworks are useful for interpreting implemented urban interventions because they distinguish short-term psychophysiological responses from longer-term behavioral and environmental changes.

But even though the policy momentum has been rapid, an acute evidence gap still exists. The majority of NBS studies have put their efforts on the environmental or governance outcomes instead of quantifiable effects on human health. Conceptual frameworks interrelate NBS to mental, physical, and social benefits, yet there is a relative lack of empirical research studies that assess implemented interventions with sufficiently strong designs that may be used to make causal inferences. The present review addresses both gaps by systematically identifying and synthesizing quantitative evaluative studies of implemented urban NBS and their health-related outcomes, using designs that support causal or exposure-based inference. In doing so, it provides an integrated picture of how forests, parks, greenways, and blue/green cooling infrastructures influence urban health, and highlights the pathways through which nature contributes to public-health resilience (Sowińska-Świerkosz and García 2022; Viti et al. 2022; Rajagopalan et al. 2024; Arriazu-Ramos et al. 2025).

## Materials and methods

This scoping review was undertaken in line with the PRISMA extension for Scoping Reviews (PRISMA-ScR, Tricco et al. 2018) and methodology outlined by the Joanna Briggs Institute (Peters et al. 2020). A scoping review approach was chosen to identify the existing evidence on the implemented urban NBS and quantifiable health related outcomes and not to aggregate effect estimates for a set of studies with a similar study design. The synthesis was structured a priori around the three-pathway model of Markevych et al. (2017), namely mitigation of environmental hazards, restoration of psychophysiological capacities, and instoration of salutogenic capacities through physical activity and social contact. This framework was selected because it explicitly links environmental exposures, psychological restoration, and behavioural or social capacity-building to health, and therefore accommodates within a single analytical structure both studies reporting direct health outcomes and studies reporting health-relevant environmental exposures that lie on the causal pathway to disease. No review protocol was registered.

### Study eligibility criteria

#### Inclusion criteria

We included primary human studies that:

- evaluated an implemented or existing urban nature-based solution (NBS), such as new or restored parks and greenways, street-tree corridors, urban forests, blue–green corridors, green roofs or walls, vegetated barriers, or constructed wetlands;
- reported at least one quantitative health-related outcome or direct health-relevant exposure pathway (e.g., mental-health indicators, physical activity or sedentary behavior, respiratory or cardiovascular outcomes, mortality or morbidity);
- used an evaluative or quasi-experimental design allowing causal or exposure-based inference — namely randomized or cluster-randomized controlled trials, controlled before–after or difference-in-differences analyses, interrupted time-series designs, or well-controlled field measurements where the NBS directly modifies a measurable exposure relevant to human health (e.g., heat load or airborne particulate concentrations);
- reported at least one quantitative health-related outcome or a direct health-relevant exposure metric with a well-established causal link to human disease — specifically heat load (UTCI), airborne particulates, or pathogenic microbial taxa — which constitute recognised intermediate endpoints on the pathway between environmental conditions and population health (Markevych et al. 2017; WHO 2021);

To focus on up-to-date evidence with policy relevance, only studies from 2015 onwards were included. We included English-language full-text articles only.

#### Exclusion criteria

We excluded studies meeting any of the following:

- were reviews, editorials, protocols, or scoping reviews;
- lacked an implemented or existing NBS intervention (e.g., cross-sectional associations between greenness and health without a defined intervention);
- relied solely on laboratory, simulated, or virtual exposures;
- reported only ecological or modeling results without field data;
- were qualitative studies without quantitative health outcomes;
- focused on non-urban settings or could not be attributed to an urban context;
- were inaccessible in full text.

### Information sources

We searched on the main database PubMed as the primary source for biomedical and public-health literature on urban NBS and health. No additional databases were searched.

### Search strategy

The full search string is reported in AppendixS1. The search combined title-level terms for urban green and blue infrastructure and NBS with title/abstract terms covering mental health, physical activity, respiratory and cardiovascular outcomes, mortality, heat, air pollution, and an urban-context filter. Non-primary article types were excluded by publication-type filter.

### Selection process

Before screening, we removed records published prior to 2015. Two reviewers independently screened titles and abstracts; disagreements were resolved through discussion and, when necessary, by a third reviewer. A two-stage screening was then performed against prespecified criteria. Title/abstract screening retained studies that addressed urban NBS and relevant health outcomes in an urban context. Full texts of potentially eligible reports were assessed for alignment with the evaluative-design criterion, the implemented NBS exposure, and the eligible outcomes. Common reasons for exclusion at full text were: study design not meeting inclusion criteria (observational or uncontrolled pre–post); ineligible exposure (not an implemented urban green/blue or NBS intervention); no eligible health or health-relevant exposure outcome; insufficient information for effect evaluation; or duplicate/overlapping populations where a more complete report was available. We present a PRISMA-ScR flow diagram with itemized exclusion reasons.

### Data collection process and data items

For each included study, we extracted the location and setting; population and sample size; type, scale, and implementation details of the NBS exposure; study design and analytic approach (e.g., randomized trial, controlled before–after, difference-in-differences, interrupted time series); follow-up period; outcome domains and measurement instruments (e.g., validated mental-health scales, physical-activity measures, or objective thermal/air-pollution metrics); effect estimates with uncertainty (e.g., odds ratios, mean differences, β-coefficients, 95% CIs); and key methodological features (covariate adjustment, clustering, or propensity methods). When multiple publications reported overlapping samples, we retained the most complete analysis. Data extraction was checked independently by a second reviewer to ensure accuracy.

### Risk of bias assessment

Given the heterogeneity of study designs, risk of bias was assessed qualitatively at the study level using a design-specific approach. No single formal standardized risk-of-bias tool was applied across all studies, because the evidence base included randomized or controlled therapeutic interventions, quasi-experimental natural experiments, longitudinal population analyses, and controlled environmental field measurements. No single validated instrument spans this range of designs; in particular, controlled environmental field measurements have no established risk-of-bias tool, and applying an instrument developed for one design to a different design would produce misleading domain ratings. We therefore used a design-specific, domain-based approach informed by the principles of established instruments, namely the Cochrane RoB 2 tool for randomized trials (Sterne et al. 2019) and ROBINS-I for non-randomized studies of interventions (Sterne et al. 2016), supplemented by exposure-assessment criteria for the environmental field measurements. Instead, we aligned the assessment domains to each study design. For randomized or controlled intervention studies, we considered randomization, allocation procedures, baseline comparability, blinding feasibility, missing data, outcome measurement, and follow-up duration. For difference-in-differences or quasi-experimental natural experiments, we considered the plausibility of parallel trends, comparator appropriateness, residual confounding, contamination, concurrent urban changes, intervention classification, and time-varying confounding. For interrupted time-series or longitudinal population analyses, we considered trend stationarity, intervention timing, ecological exposure assignment, and secular changes. For controlled environmental field measurements, we considered site selection, comparator appropriateness, exposure-measurement validity, temporal representativeness, and the indirectness of health inference where direct clinical outcomes were not measured. We also considered exposure misclassification for distance- or area-based definitions and self-report bias for behavioral or mental-health outcomes. Assessments were performed independently by two reviewers and reconciled by consensus. Each study was assigned an overall qualitative judgement by aggregating these domain ratings: low risk of bias when no domain raised concerns, some concerns when one or more domains raised non-critical concerns, and high or some concerns when a domain likely to bias the result was present. The main reasons are summarized in Table 2.

### Synthesis methods

We summarized findings narratively, grouping studies by NBS type and outcome domain and noting the direction and size of effects where comparable.

## Results

### Study selection

**Fig. 1:**
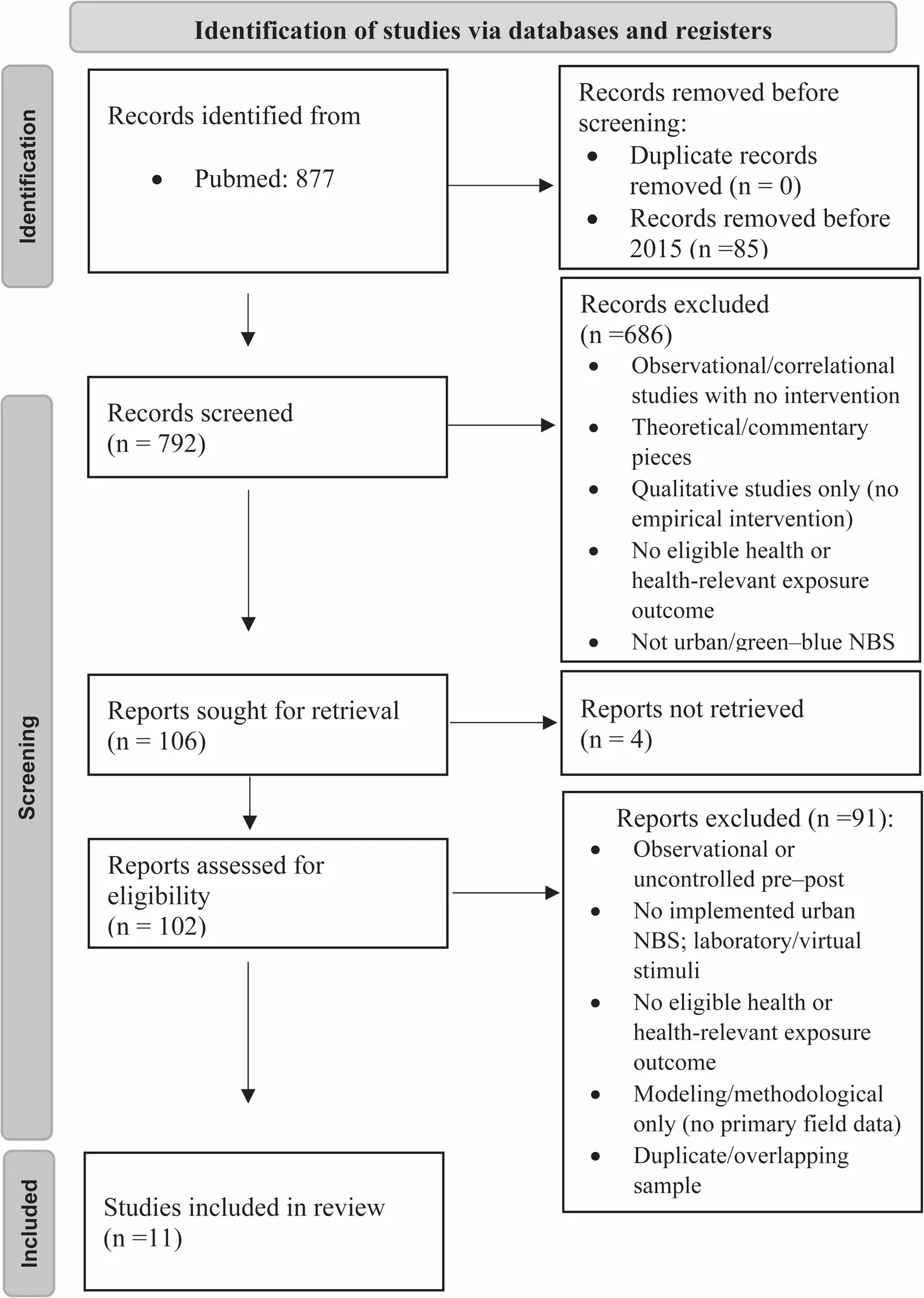
PRISMA-ScR flow diagram showing identification, screening, eligibility and inclusion of studies

The database search identified 877 records in PubMed. Before screening, we removed items published prior to 2015 (*n* = 85), leaving 792 records for title/abstract screening. Of these, 686 were excluded (most commonly observational studies without an implemented intervention, theoretical/commentary pieces, qualitative-only reports, or records without a health outcome). We sought 106 full texts; four could not be retrieved. We assessed 102 reports for eligibility and excluded 91 at full text. The most common reasons were ineligible study design (observational or uncontrolled pre–post), lack of an implemented urban NBS/BGI intervention (including laboratory or virtual exposures), absence of an eligible health or health-relevant exposure outcome, modelling-only evidence without primary field data, duplicate or overlapping samples, and insufficient information for effect evaluation. After full-text assessment, eleven studies met all criteria and were included in the review (Fig. 1).

The studies met the inclusion criteria are covering a range of urban nature-based solutions (NBS) such as urban forests, parks, greenways, blue spaces, and hybrid green–blue systems. The 11 studies covered three broad intervention families. First, three studies examined structured therapeutic exposure programs delivered in urban forests or parks. These were retained because they quantified the health effects of real-world exposure to urban nature, but they are interpreted cautiously as NBS-enabled health programs rather than evaluations of newly built NBS infrastructure. Second, four studies evaluated greenways, blue-green corridors or park expansion as public-realm interventions affecting activity, safety, mortality or childhood overweight. Third, four studies evaluated vegetated barriers, trees, green roofs, blue-green cooling sites or wetland/blue-green restoration as exposure-mitigation infrastructure. Table 1 summarizes the main study characteristics and quantitative findings. All studies reported quantitative results relevant to public health—either through direct environmental measurements or population health outcomes. The results were synthesized into two primary streams of evidence to make it easier to interpret the results. The former comprised experimental or field-based research assessing the quantifiable effects of NBS on environmental conditions in a close relation to health, including regulation of temperature, quality of air, and elimination of pollution. The second category included epidemiological and population-based studies which related the exposure to urban greenery or blue spaces to the health effects, such as mortality, cognitive performance, and mental well-being. However, collectively these studies demonstrate how the various types of urban nature may have direct or indirect effects on human health by acting complementary to one another.

**Table 1:** Summary of included studies evaluating the health impacts of urban nature-based solutions (PICO)

Study	Location /Setting	Population	Intervention (Nature-Based Solution)	Study Design	Comparator / Control	Key Instruments	Outcome	Main findings
Yeon et al. 2022	South Korea; urban forest/park therapy	adults with depression (*N* = 89)	Guided sessions in an urban forest/park	Randomized controlled trial	Indoor relaxation group	PHQ-9, GAD-7, PSS	Stress, mood, anxiety	Lower stress and anxiety vs. control
Yeon et al. 2023a, b (November)	South Korea; urban forest	Office workers with depressive symptoms (*N* = 62)	App-guided park visits and nature mindfulness	Controlled before–after (personal baseline)	Personal baseline (before the program)	PSS, PSQI, WHO-5	Stress, wellbeing, sleep quality	Better sleep and wellbeing after program
Yeon et al. 2023a, b (October)	South Korea; urban park	City residents with depression (*N* = 74)	Repeated walks in a local park	Controlled before–after (personal baseline)	Personal baseline (before the program)	PHQ-9, WHO-5	Mental wellbeing, depression	Lower depression after exposure
Hunter et al. 2021	Belfast, UK; residential greenway	Residents near new greenway (*N* = 467)	New planted greenway for walking and cycling	Controlled before–after (PARC study)	Pre-intervention baseline	Accelerometry, self-report surveys	Physical activity, perceived safety	Increased walking and cycling frequency; improved safety perception.
Auchincloss et al. 2019	Philadelphia USA; greenway	Commuters and neighborhood residents (*N* = 1,284 counts)	Upgraded shaded greenway with vegetation	Natural experiment (before–after pedestrian counts)	Baseline counts prior to implementation	Pedestrian counters, surveys	Active transport, social interaction	Higher pedestrian counts; greater community use of public space
Tieges et al. 2020	UK; / blue–green corridor	Urban population;	Restored canal / blue–green corridor	17-year longitudinal quasi-experiment (DiD)	Non-restored urban districts	Registry mortality data	All-cause mortality	Lower mortality and smaller gaps between areas
Koebe 2025	Berlin, Germany; urban districts	School-entry children (*N* ~ 12,000)	Large new public park and surrounding green area	Difference-in-differences (10-year panel)	Districts without new park	Health registry BMI data	Overweight prevalence	Slower increase in overweight
Khan et al. 2025	Lithuania; traffic road	Pedestrians near traffic (field measurement)	Vegetated roadside hedge	Controlled field measurement	Roadside with no hedge	Particle counters, SEM analysis	Airborne microplastic levels	40–60% fewer particles behind hedge
Wang et al. 2025	Xiong, China; newly built district	City residents	Newly created wetlands / blue–green restoration	Controlled before–after (soil sampling)	Same area before restoration	16 S rRNA sequencing, metagenomics	Soil microbes, potential pathogens	Fewer potential pathogens after restoration
Sahani et al. 2023	Residents in hot areas;	Multiple cities	Vegetated / blue–green urban sites	Controlled field measurements	Nearby paved sites	Weather stations, thermal sensors	Local temperature	2–6 °C cooler in vegetated areas
Anderson and Gough 2022	Toronto, Canada; City neighborhoods;	Multiple cities	Trees, green roofs, park vegetation	Multi-site controlled field measurement	Built-up surfaces without vegetation	Radiometric sensors, portable weather stations	Surface / air temperature	3–5 °C cooler in vegetated areas

In order to compile such a literature in a coherent way, the identified literature was categorized based on the mechanism of health impact predominant in each study, but not on geography or the type of vegetation. To organize the included literature in a coherent way, studies were categorized inductively according to the predominant health-impact mechanism, drawing on the three-pathway model of Markevych et al. (2017): (1) psychophysiological restoration — interventions in which structured exposure to nature directly affects mental health and stress recovery; (2) behavioral activation and health equity — landscape-scale NBS that facilitate physical activity, social engagement, and reduce health disparities; and (3) environmental exposure mitigation — vegetated or blue-green elements that reduce pollutants, pathogens, or heat stress.

The organization permits a mechanistic understanding of the role of urban NBS in promoting the human health -fitting psychological, behavioral and biophysical contexts into a single scheme.

### Psychophysiological restoration

The psychological well-being of the exposure to nature was investigated in three clinical and quasi-experimental interventions within the urban forest and park settings (Yeon et al. 2022, 2023a, b November; Yeon et al. 2023a, b October). The participants were urban dwellers who had quantifiable symptoms of stress or anxiety. Park-based sessions or forest-therapy sessions were involved in slow walking, breathing in a controlled manner and practicing mindfulness. Two of them were based on randomized or controlled design (Yeon et al. 2022, 2023a, b November) and one on a controlled clinical comparison (Yeon et al. 2023a, b; October) which guarantee a high internal validity.

All interventions demonstrated a significant reduction of the perceived stress and anxiety scores and a raise in mood and better sleep quality compared to controls. In the mobile-based program (Yeon et al. 2023a, b November), the same was used with digital guidance, which denotes the possibility of scaling. These findings lend cautious support to the view that urban forests and parks may function as health-supportive settings when embedded within structured therapeutic or restorative interventions, while highlighting the potential contribution of NBS to mental-health promotion.

### Behavioral activation and health equity

Four articles explored built nature-based infrastructures that were aimed at modifying daily movement and social accessibility: recently introduced or refurbished greenways (Auchincloss et al. 2019; Hunter et al. 2021), a reconstructed canal and blue-green corridor (Tieges et al. 2020), and the creation of a big urban park in Berlin (Koebe 2025). They both used before-after or quasi-experimental design, which reinforced causation inference.

The greenway initiatives in the two instances (Auchincloss et al. 2019; Hunter et al. 2021) reported significant gains in walking and cycling, sense of safety, and neighborhood satisfaction after implementation. These behavioral changes were not confined to recreation, but they denoted better access and inclusiveness of the public space.

On the metropolitan level, the canal restoration project (Tieges et al. 2020) was associated with better blue-green connectivity and mortality decrease and narrowing mortality disparities among socio-economic groups. Likewise, the Berlin park study (10 years) (Koebe 2025) found that an access to new green-space was related to a reduced increase in childhood overweight prevalence than control districts.

Collectively, these findings imply that infrastructural NBS may facilitate active living and, at the same time, serve as a tool of urban health equity that can change the morbidity rates of populations, and not just individuals.

### Environmental exposure mitigation

The last category of studies evaluated NBS that was mainly developed to protect the environment but showing the quantifiable health impact.

An installed vegetated hedge of Thuja occidentalis (at a traffic route) decreased the airborne deposition of microplastics in the region of the barrier by up to 60%, including fragments and fibers formed during tire and brakes wear (Khan et al. 2025). Such field evidence can highlight the importance of green buffers to reduce the exposure to inhalable pollution in heavily populated cities even in a small scale.

In Xiong, China, a massive restoration of wetlands and blue-green network (Wang et al. 2025) had a positive impact on soil microbial diversity, decreasing the number of potentially pathogenic taxa, and it has used secondary public-health co-benefits through the stabilization of the ecosystem.

The thermal environment in vegetated and impervious urban surfaces was compared and contrasted in two other field measurements (Anderson and Gough 2022; Sahani et al. 2023). In several of the heat waves, the temperature was lower by 2–6 °C at trees or green roofs or blue-green corridors compared with neighboring built-up areas-differences that were large enough to reduce the risk of human heat-stress. Taken together, these works show that NBS can be effective as protective structures, filtering agents, stabilizing the microbiome of an urban environment, and regulating thermal extremes. Four studies measured reductions in health-relevant environmental exposures (heat load, airborne microplastics, and pathogenic soil taxa) rather than downstream clinical outcomes. These exposures are not health outcomes in themselves; they are established upstream determinants on the mitigation pathway, with well-documented causal links to morbidity and mortality, and were therefore retained as health-relevant exposure proxies rather than as direct measures of health status (Markevych et al. 2017; WHO 2021).

A brief summary of the evidence base will be presented in Table 1 to provide the main characteristics of the eleven studies satisfying all the inclusion criteria. The table is structured in the PICO format (Population, Intervention, Comparator, Outcome) that enables the easy comparison of the heterogeneous designs. It emphasizes that all the studies used were in real-life urban settings, applied to nature-based interventions that were measurable, and evaluated the health or exposure-related outcomes in quantitative ways.

The design-specific qualitative risk-of-bias assessment for each included study is presented in Table 2. Most studies were judged as having some concerns rather than low risk of bias. The most frequent reasons were limited follow-up, lack of feasible blinding, possible residual confounding, exposure misclassification, reliance on self-reported outcomes, and indirectness where environmental exposure metrics rather than direct health outcomes were measured. A rating of some concerns should be read as indicating that a study is informative for the specific short-term effect or exposure–response relationship it evaluated, but that, taken on its own, it provides only limited support for strong causal claims about long-term clinical outcomes. Consistent with the mapping purpose of a scoping review, the judgements in Table 2 are intended to signal where the evidence is more or less secure rather than to rank or exclude studies.

**Table 2:** Qualitative risk-of-bias assessment by study design

Study	Overall judgment	Main reasons for judgement
Yeon et al. 2022	Some concerns	The controlled design supports short-term inference, but follow-up was limited, participant blinding was not feasible, outcomes relied largely on self-reported or clinical scales, and the therapeutic programme design makes it difficult to isolate the effect of the urban nature setting from the guided intervention.
Yeon et al. 2023a, b (November)	Some concerns	Randomization strengthens internal validity; however, the small sample size, limited follow-up, lack of feasible participant blinding, and reliance on mental-health scales introduce some uncertainty.
Yeon et al. 2023a, b (October)	High/some concerns	The pre–post design limits causal attribution, as changes may reflect temporal trends or participant expectations. In addition, the app-guided therapeutic component was evaluated in existing urban forests rather than as a newly implemented NBS intervention.
Hunter et al. 2021	Some concerns	The natural-experiment design and use of paired comparison locations strengthen inference, but residual confounding, secular trends, and concurrent neighbourhood changes cannot be fully excluded.
Auchincloss et al. 2019	Some concerns	The use of comparison areas improves causal interpretation, but exposure uptake varied among residents, behavioural outcomes may be influenced by self-report or measurement limitations, and concurrent community changes may have affected the results.
Tieges et al. 2020	Some concerns	The long follow-up and population-level data strengthen the analysis; however, ecological exposure assignment, possible exposure misclassification, and concurrent regeneration or climate-adaptation measures may have contributed to the observed associations.
Koebe 2025	Low/some concerns	The quasi-experimental design and synthetic-control approach strengthen causal inference. Remaining concerns relate mainly to district-level exposure classification, which may not fully capture individual access to or use of the new green space.
Khan et al. 2025	Some concerns	Controlled field measurements provide direct evidence of exposure reduction, but the assessment was limited to environmental microplastic indicators and did not measure individual exposure, dose, or clinical health outcomes.
Wang et al. 2025	Some concerns	Field-based microbial measurements are relevant to potential exposure pathways, but the public-health interpretation remains indirect because no individual exposure assessment or clinical health endpoints were reported.
Sahani et al. 2023	Some concerns	Field measurements support the cooling effect of green–blue interventions, but health benefits were inferred from thermal exposure metrics rather than directly measured through physiological or clinical outcomes.
Anderson and Gough 2022	Some concerns	Cooling potential was assessed in real urban settings, but the study did not directly link temperature reductions to individual heat exposure, physiological response, morbidity, or mortality outcomes.

### Limitations

The research was restricted to PubMed, which indexes biomedical and public-health literature. This choice was consistent with the public-health scope of the review but means that studies indexed primarily in urban planning, environmental engineering, or landscape architecture databases were not captured. The review should therefore be read as a scoping synthesis of the biomedical evidence base on urban NBS and health, not as a comprehensive map of all implemented NBS interventions across disciplines. Four included studies report reductions in health-relevant exposures — heat load, airborne microplastics, and pathogenic microbial taxa — rather than clinical outcomes. These two evidence types are treated as analytically distinct throughout the Results and Discussion.

## Discussion

This scoping review set out to map whether, and through which pathways, implemented urban nature-based solutions (NBS) and blue-green infrastructure (BGI) are associated with measurable health-relevant effects. Across the eleven studies meeting our inclusion criteria, effects were observed along all three pathways of the Markevych et al. (2017) framework: improvements in psychological wellbeing (restoration); increases in health-supporting behaviours and reductions in health disparities (instoration or behavioural activation); and reductions in health-relevant environmental exposures (mitigation). The first two pathways were captured through direct health outcomes, whereas the third was captured through upstream environmental exposures rather than clinical endpoints. Framing the findings in this way allows NBS and BGI to be positioned as intentional public-health interventions rather than incidental green amenities.

Previous syntheses on urban greenness and health have primarily relied on observational contrasts between greener and less green neighbourhoods (Bratman et al. 2019; Markevych et al. 2017). These studies have revealed favourable associations with stress, self-rated health, and cardiometabolic risk, but leave two fundamental issues unresolved: the direction of causality, and the treatment of urban nature as a static background feature rather than a designed intervention. By contrast, the studies included here evaluated specific interventions compared against defined baselines or control areas — an evaluative approach that moves the evidence base substantially closer to causal inference.

Three substantive findings emerged. First, consistent mental-health benefits were observed following structured exposure to urban green spaces (Yeon et al. 2022, 2023a, b October; Yeon et al. 2023a, b November). These effects appeared within short time frames (weeks to months), consistent with prior experimental work suggesting that restorative responses to nature can occur rapidly (Bratman et al. 2019). However, these studies evaluate therapeutic programmes delivered in urban green settings rather than the NBS infrastructure per se; whether comparable benefits would accrue from unguided access to the same spaces remains an open question. Long-term persistence of mental-health gains was not examined.

Second, landscape-scale interventions operated on health outcomes at the population level rather than solely at the individual level (Auchincloss et al. 2019; Tieges et al. 2020; Hunter et al. 2021; Koebe 2025). This distinction is important: mental-health benefits from structured sessions may be detectable only in controlled studies, whereas behavioral and mortality outcomes accumulated over years can be captured in registry data and reveal whether NBS narrow health inequalities across socioeconomic groups. It is worth noting that all health effects ultimately occur in individuals; the concept of ‘population-level’ outcomes here refers to the scale of measurement and the detectability of effects through population surveillance rather than to a fundamentally different biological process. The magnitude and duration of effects in this pathway suggest that physical reconfiguration of urban space can alter health trajectories in disadvantaged populations over timescales of years to decades.

Third, several NBS and BGI interventions functioned as protective health infrastructure by directly reducing pathogenic and physicochemical exposures (Anderson and Gough 2022; Sahani et al. 2023; Khan et al. 2025; Wang et al. 2025). The temperature reductions of 2–6 °C documented in vegetated versus built areas align with established thresholds for heat-stress mitigation; the approximately 40–60% reduction in microplastic particle deposition behind a roadside hedge is notable given growing evidence of respiratory and systemic effects of inhaled microplastics. These findings suggest that even modest, site-specific NBS may deliver measurable exposure reductions in high-traffic urban environments.

Considered together, these three strands converge on the same conclusion: urban NBS act on physiology (reducing stress and improving sleep), on behavior and social access (making outdoor activity safer and more equitable), and on environmental exposure (lowering heat, pollutants, and microbial risk). This multi-pathway architecture helps explain both the policy appeal of NBS and the challenges of evaluating them in a standardized way, since different urban agencies tend to monitor different benefit types in isolation.

**Fig. 2:**
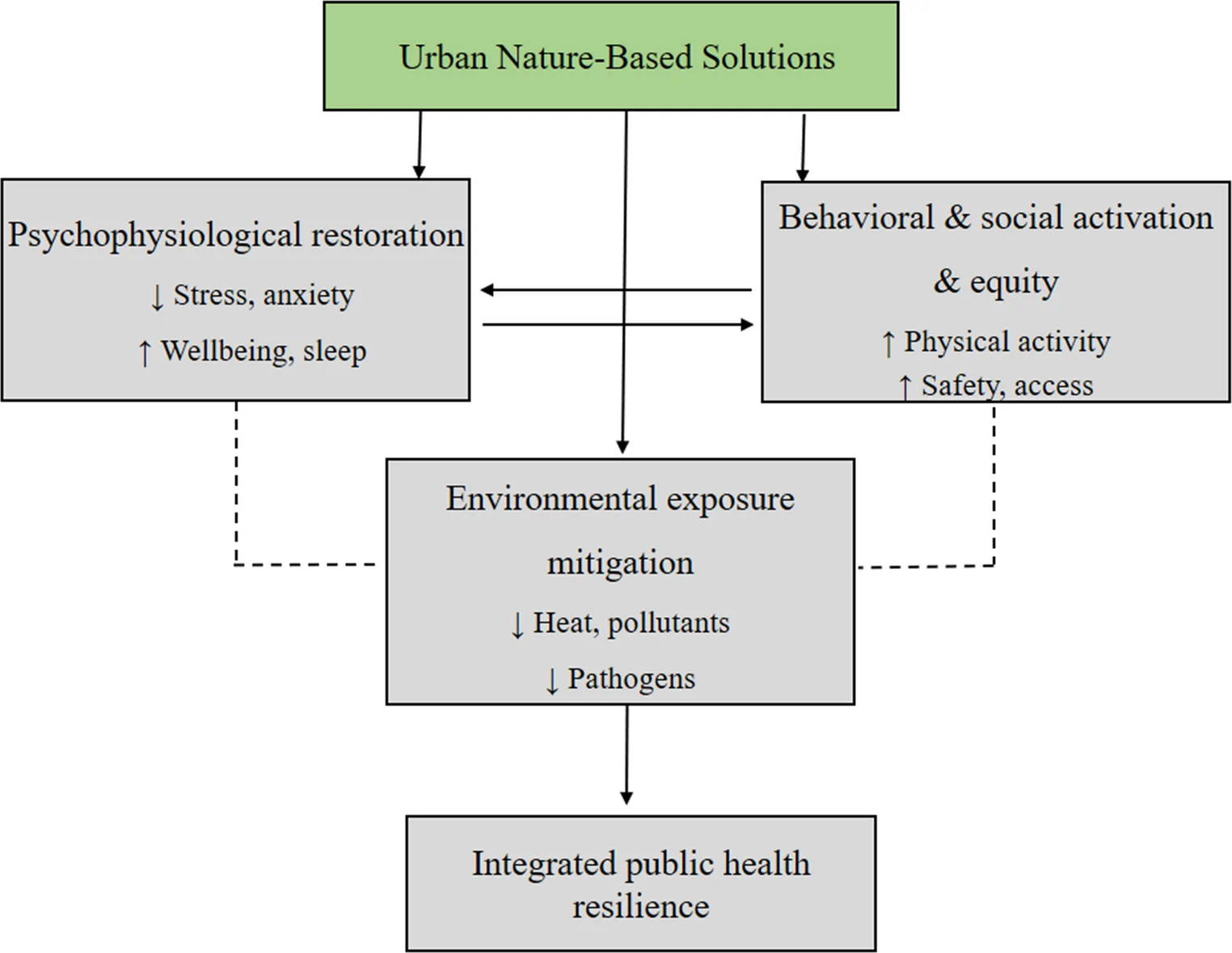
Conceptual model showing interacting pathways through which urban nature-based solutions (NBS) influence human health

Figure 2 presents a conceptual model, adapted from the three-pathway framework of Markevych et al. (2017), that summarises the routes through which the included interventions appear to influence health: psychophysiological restoration, behavioural and social activation, and mitigation of environmental exposures. The model distinguishes (1) restoration, comprising short-term psychophysiological recovery, stress reduction and improved sleep; (2) instoration or behavioural capacity-building, comprising physical activity, social contact, perceived safety and equitable access; and (3) mitigation, comprising reduction of environmental stressors such as heat, air pollutants, microplastics and microbial hazards. Rather than being newly derived from this review, these pathways are drawn from an established framework and are supported by the studies synthesised here. The pathways are not mutually exclusive and may reinforce one another, collectively supporting integrated public-health resilience at the city scale, which makes the model a policy-relevant basis for integrating NBS and BGI into urban public-health planning.

NBS simultaneously trigger psychophysiological restoration (reducing stress and anxiety, improving wellbeing and sleep), behavioral and social activation and equity (increasing physical activity, safety, and access), and environmental exposure mitigation (reducing heat, air pollutants, and pathogens). These pathways reinforce one another through secondary feedback loops (dashed arrows), collectively supporting integrated public-health resilience in urban environments.

## Conclusions

This scoping review provides evidence that implemented urban NBS and blue-green infrastructure can function as active public-health infrastructure, operating through three complementary pathways: psychophysiological restoration, behavioral activation and health equity, and environmental exposure mitigation. The focus on implemented, evaluated interventions with defined comparators strengthens the policy relevance of this evidence. In the context of rising urban heat, air pollution, and mental-health pressures, these findings support the integration of NBS into mainstream public-health planning — provided that future projects embed health-focused monitoring from the outset.

Future research should therefore prioritize longitudinal and quasi-experimental designs, standardized exposure assessment, and collaboration across environmental science, public health and urban design so that the multiple pathways identified here can be quantified more systematically.

Overall, in the context of rising urban heat, air pollution and mental-health pressures, the evidence synthesized in this review supports the integration of NBS into mainstream public-health planning. While the current evidence base is still modest, it points to a practical conclusion: well-implemented urban nature can make cities not only greener, but also measurably healthier, more equitable and more resilient.

## Data Availability

No datasets were generated or analysed during the current study.

## Appendix S1. Full PubMed search string

The following search string was run in PubMed on November 2025. No date or language restrictions were applied beyond the 2015 publication-year filter applied during screening.

((“greenspace“[Title] OR “green space“[Title] OR “urban green*“[Title] OR “urban park*“[Title] OR “urban forest*“[Title] OR “street tree*“[Title] OR “greenway*“[Title] OR “blue space*“[Title] OR “green infrastructure“[Title] OR “nature-based solution*“[Title] OR “green roof*“[Title] OR “green wall*“[Title] OR “constructed wetland*“[Title]) AND (“public health“[Title/Abstract] OR “mental health“[Title/Abstract] OR depression[Title/Abstract] OR anxiety[Title/Abstract] OR “quality of life“[Title/Abstract] OR “physical activ*“[Title/Abstract] OR exercise[Title/Abstract] OR respiratory[Title/Abstract] OR cardiovascular[Title/Abstract] OR mortality[Title/Abstract] OR morbidity[Title/Abstract] OR heat[Title/Abstract] OR heatwave*[Title/Abstract] OR UTCI[Title/Abstract] OR “air pollution“[Title/Abstract] OR PM2.5[Title/Abstract] OR NO2[Title/Abstract] OR ozone[Title/Abstract] OR diabetes[Title/Abstract] OR “birth outcome*“[Title/Abstract] OR prenatal[Title/Abstract]) AND (urban[Title/Abstract] OR city[Title/Abstract] OR cities[Title/Abstract])) NOT (Review[Publication Type] OR Editorial[Publication Type] OR Protocol[Title/Abstract] OR Scoping[Title/Abstract])

This search returned 877 records. Records published before 2015 (*n* = 85) were removed before screening, leaving 792 records for title/abstract review.

## Declarations

### AI use statement

No artificial-intelligence or large-language-model tools were used at any stage of this work, including study selection, screening, data extraction, analysis, interpretation, or manuscript preparation.
